# Safety and Efficacy of Transoral Robotic Thyroidectomy for Thyroid Tumor: A Systematic Review and Meta-Analysis

**DOI:** 10.3390/cancers14174230

**Published:** 2022-08-31

**Authors:** Yun Jin Kang, Jin-Hee Cho, Gulnaz Stybayeva, Se Hwan Hwang

**Affiliations:** 1 Department of Otolaryngology-Head and Neck Surgery, Yeouido St. Mary’s Hospital, College of Medicine, The Catholic University of Korea, Seoul 06591, Korea; 2 Department of Physiology and Biomedical Engineering, Mayo Clinic, Rochester, MN 55905, USA; 3 Department of Otolaryngology-Head and Neck Surgery, Bucheon St. Mary’s Hospital, College of Medicine, The Catholic University of Korea, Seoul 06591, Korea

**Keywords:** transoral thyroidectomy, robotic thyroidectomy, transoral approach, vestibular approach, transoral-vestibular robotic thyroidectomy, thyroid neoplasm, meta-analysis

## Abstract

**Simple Summary:**

This systemic review and meta-analysis compared and analyzed the safety and effectiveness of transoral robotic thyroidectomy on the thyroid tumor with other thyroid approaches. Transoral robotic thyroidectomy showed similar results to other robotic-assisted thyroid surgeries. Compared to a conventional open thyroidectomy, transoral robotic thyroidectomy had longer operational times and hospitalization days, and worse postoperative pain, but a higher cosmetic satiation score. However, more randomized controlled studies need to be included and analyzed.

**Abstract:**

Background: To assess the safety and effectiveness of transoral robotic thyroidectomy (TORT) in thyroid tumor. Methods: PubMed, Embase, Web of Science, SCOPUS, Cochrane database, and Google Scholar up to June 2022. Studies comparing outcomes and complications between TORT and control groups (robotic bilateral axillo-breast, trans-axillary, postauricular approach, conventional open thyroidectomy (OT), and transoral endoscopic approach) were analyzed. Results: Ten studies of 1420 individuals. The operative time (SMD 1.15, 95%CI [0.48; 1.89]) was significantly longer and the number of retrieved lymph nodes (LNs) (SMD −0.27, 95%CI [−0.39; −0.16]) was fewer in TORT than in the control group. The postoperative cosmetic satisfaction score (SMD 0.60, 95%CI [0.28; 0.92]) was statistically higher in TORT than in the control group. In subgroup analysis, there was no significant difference between robotic surgeries. However, TORT had significantly longer operative times (SMD 2.08, 95%CI [0.95; 3.20]) and fewer retrieved LNs (SMD −0.32, 95%CI [−0.46; −0.17]) than OT. TORT satisfied significantly more patients in cosmetic view. However, it increased hospitalization days and postoperative pain on the operation day and first day compared to OT. Conclusions: TORT is not inferior to other robotic-assisted approaches. Its operation time and hospitalization days are longer and postoperative pain is greater than OT, although its cosmetic satisfaction is high.

## 1. Introduction

The prevalence of thyroid tumors, including thyroid cancer, has been increasing for several decades [1]. With the development of diagnostic tools for small thyroid lesions, thyroid surgery from lobectomy to total thyroidectomy has increased [2,3]. In the past, the standard treatment for thyroid tumors was conventional open thyroidectomy (OT) [4]. Although conventional OT exposes the thyroid gland sufficiently, it can leave a large scar on the neck [5]. Most patients with thyroid nodules are women. About 5% of females have thyroid nodules [1]. Most patients with thyroid tumors, especially young female patients, are concerned about neck scars [6,7]. Various endoscopic and robotic thyroid surgeries have been studied for decades to avoid anterior neck incision [4,5].

Since 2000, surgical techniques for endoscopic thyroid surgery [8] and minimally invasive video-assisted thyroidectomy [9] have been introduced. Since then, various surgical methods such as the robotic bilateral axillo-breast approach (BABA), robotic postauricular approach (PAA), and transoral endoscopic approach via vestibular approach (TOETVA) have been developed [5,10,11,12]. Among them, TOETVA is the only technique that does not cause visible scarring on the skin [13,14,15]. In 2007, da Vinci robot-assisted thyroid surgery was introduced [16]. Transoral robotic thyroidectomy (TORT) was introduced later [17,18]. It was found to be safe and effective in achieving functional and oncological outcomes [17]. In addition, it is more useful for identifying central lymph nodes than other thyroid surgery [17,18,19,20,21,22]. Robotic thyroid surgery using a trans-axillary approach is one of the most popular robotic techniques [21]. Conventional OT has already demonstrated excellent results for a long time. However, robotic-assisted thyroid surgery is relatively controversial [23].

Thus, the objective of this study was to perform a meta-analysis to evaluate the safety and effectiveness of TORT for thyroid tumor compared to traditional OT, robotic BABA, and TOETVA for operative outcomes, postoperative outcomes, and complications. To the best of our knowledge, this is the first meta-analysis that directly compares TORT with other approaches for thyroid tumors.

## 2. Materials and Methods

### 2.1. Search Strategy

**Figure 1:**
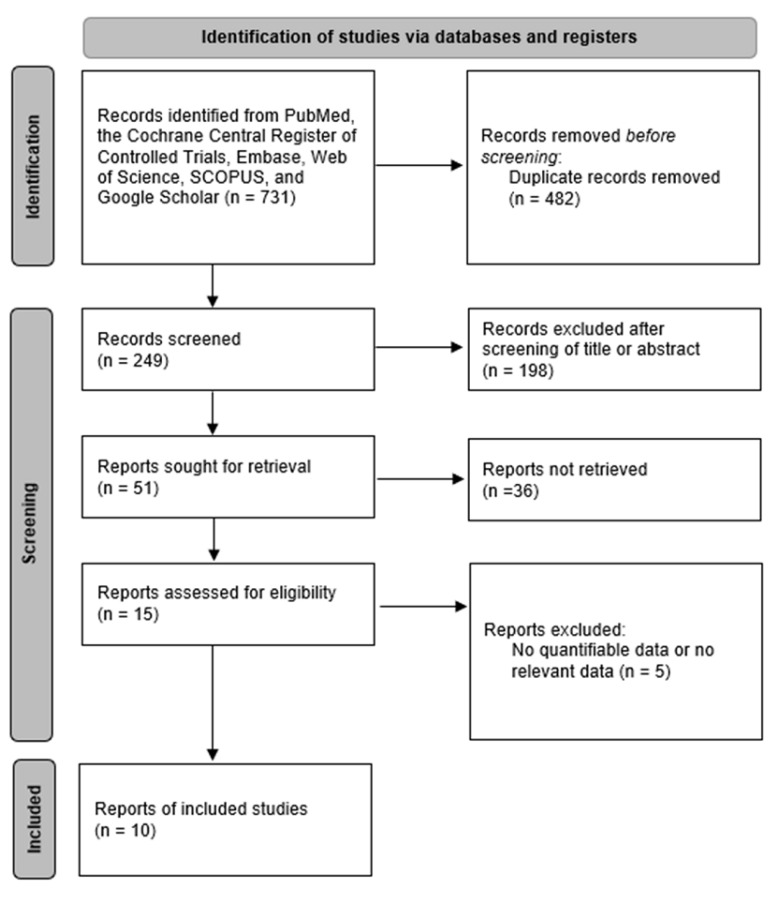
Diagram of selection of studies.

PubMed, SCOPUS, Embase, the Web of Science, Google Scholar, and the Cochrane database were searched to retrieve prospective or retrospective articles published in English prior to June 2022. Thyroidectomy, transoral thyroidectomy, remote-access thyroid surgery, minimally invasive surgery, robotic thyroidectomy, robotic thyroid surgery, surgical approaches, transoral approach, vestibular approach, transoral-vestibular robotic thyroidectomy, bilateral axillo-breast approach, open thyroidectomy, thyroid neoplasm, thyroid carcinoma, thyroid nodule, cosmesis, and comparison were used as search terms and keywords. Two independent literature reviewers reviewed and screened titles and abstracts of all searched studies, excluding studies about patients with thyroid neoplasm, not related to TORT. If the abstract alone could not determine whether or not the study should be included, the full text was then checked to determine its eligibility. Figure 1 presents a flow chart for selecting eligible studies to be analyzed. We registered the study protocol on Open Science Framework (https://osf.io/gtnck/) (accessed on 25 July 2022).

### 2.2. Data Extraction and Risk of Bias Assessment

Data were extracted from selected eligible studies regarding the number of patients, scale and score used for cosmetic satisfaction, operative time, hospitalization day, postoperative pain score, retrieved lymph node (LN) number, incidence or percentage of postoperative bleeding, skin flap perforation, postoperative hypoparathyroidism, vocal cord palsy, incidental parathyroidectomy, seroma, and *p*-value in comparison between treatment (TORT) group and control group (other types of thyroidectomy, including robotic BABA, robotic PAA, conventional OT, TOETVA). Data were then organized using a standardized format [17,18,19,21,24,25,26,27,28,29,30]. The Newcastle–Ottawa Scale was used to evaluate non-randomized controlled studies [31,32].

### 2.3. Statistical Analysis

Meta-analyses were conducted using ‘R’ statistical software (R Foundation for Statistical Computing, Vienna, Austria). When representing the original data as a continuous variable, meta-analysis was performed using standard mean difference (SMD). For all other cases, incidence analyses were performed using odds ratio (OR). Sensitivity analyses were also conducted to assess the effect of each study on overall meta-analysis results.

## 3. Results

**Figure 2:**
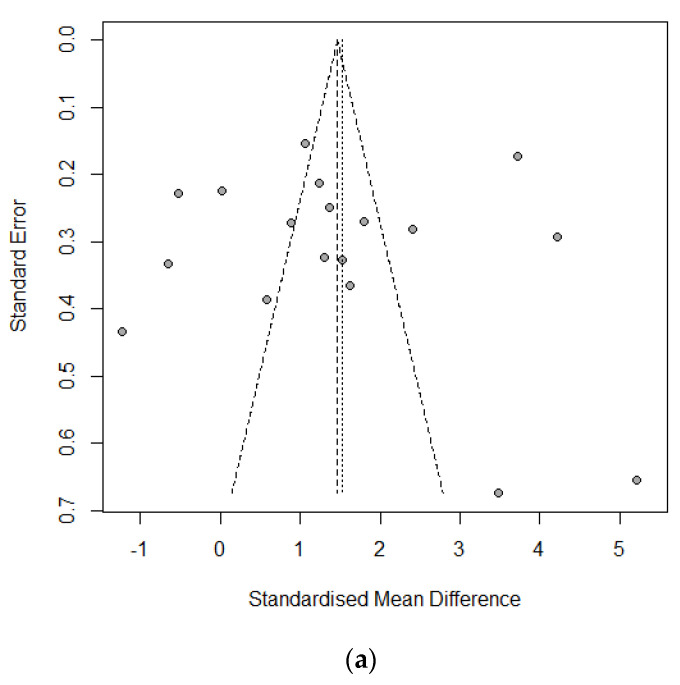

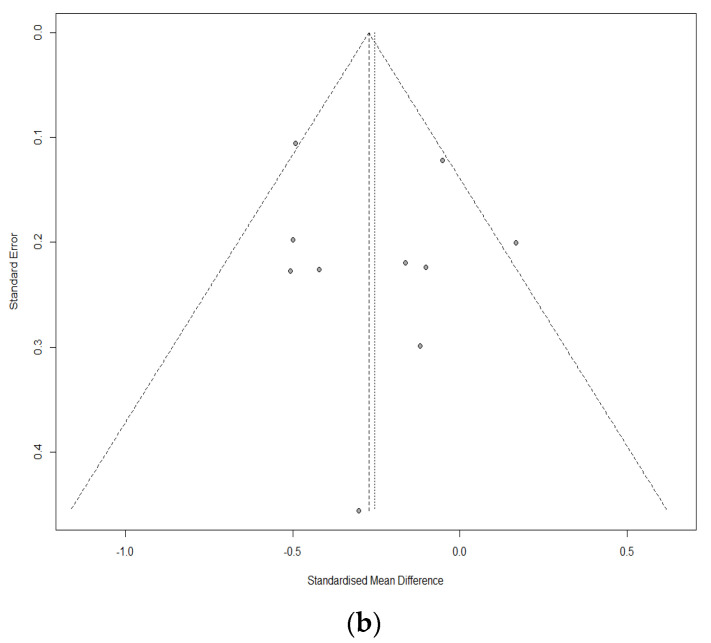

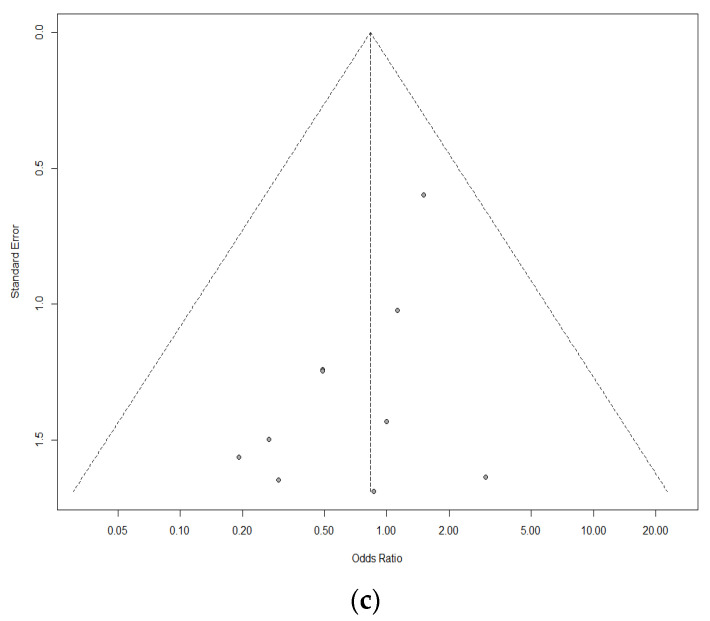
Funnel plot for publication bias of operative time (**a**), retrieved lymph node number (**b**), and temporary vocal cord palsy (**c**).

Ten studies of 1420 individuals were included. Study characteristics are summarized in Table 1. Egger’s test and Begg’s funnel plot analyses revealed no publication bias for operative time (OR 0.4171), retrieved LN number (OR 0.6468), or incidence of temporary vocal cord palsy (OR 0.19) in the included studies (Figure 2). In contrast, the number of included studies for cosmetic satisfaction, hospital day, pain score, and incidence of bleeding, flap perforation, parathyroidectomy, seroma, permanent hypoparathyroidism, and vocal cord palsy was insufficient to adequately obtain a funnel plot or perform advanced regression-based assessments. Thus, publication bias was not evaluated for them.

### 3.1. Operation-Related Outcomes between Treatment Group and Control Group

**Figure 3:**
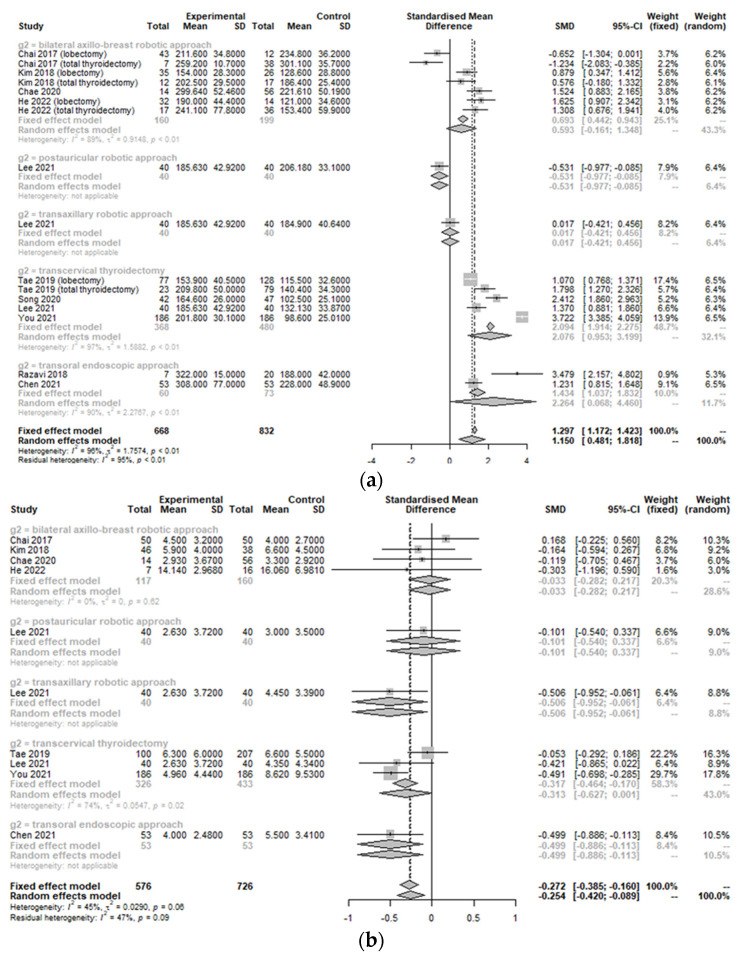
Forest plot for standard mean difference in operation-related outcomes (operative time (**a**) and retrieved lymph node number (**b**)) (total: number of participants per group).

The operative time (SMD 1.15, 95% CI [0.48; 1.82]; I^2^ = 96%) was significantly longer and the retrieved LN number (SMD −0.27, 95% CI [−0.39; −0.16]; I^2^ = 45%) was fewer in the TORT group than in the control group (Figure 3). There was no significant difference in the positive LN number (SMD −0.0858, 95% confidence interval (CI) [−0.3441; 0.1725]; I^2^ = 66.7%), incidence of skin flap perforation (OR 6.0165, 95% CI [0.9318; 38.8464]; I^2^ = 0.0%), or incidental parathyroidectomy (SMD 1.3392, 95% [0.3981; 4.5049]; I^2^ = 0.0%) between the treatment group and the control group (Table 2).

There was a significant heterogeneity (I^2^ > 50%) in operation-related measurements because control groups involved different types of approaches. Therefore, subgroup analysis was conducted to assess the change in comparative advantage or disadvantage of TORT according to the type of operation. In subgroup analysis, there were no significant differences in some operation-related measurements between robotic surgeries (TORT, trans-axillary robotic approach, and robotic PAA). However, the TORT group had significantly longer operative times and fewer LNs retrieved than the conventional OT group (SMD 2.08, 95% CI [0.95; 3.20]/SMD −0.32, 95% CI [−0.46; −0.17]) and the TOETVA group (SMD 2.26, 95% CI [0.07; 4.46]/SMD −0.50, 95% CI [−0.89; −0.11]). The TORT group had significantly longer operative times than the robotic BABA group (SMD 0.59, 95% CI [−0.16; 1.35]).

### 3.2. Peri-Operative Complications between the Treatment Group and Control Group

**Figure 4:**
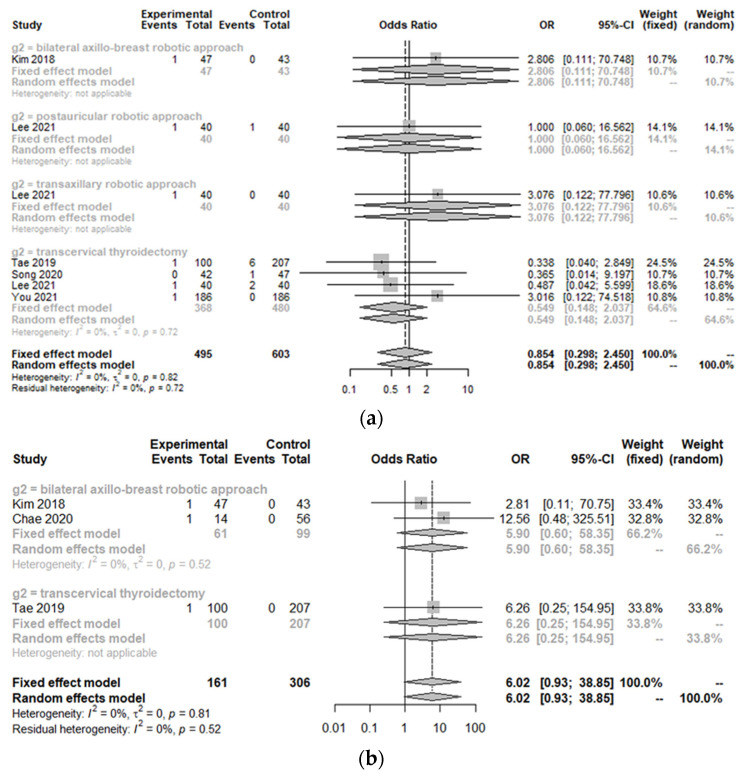

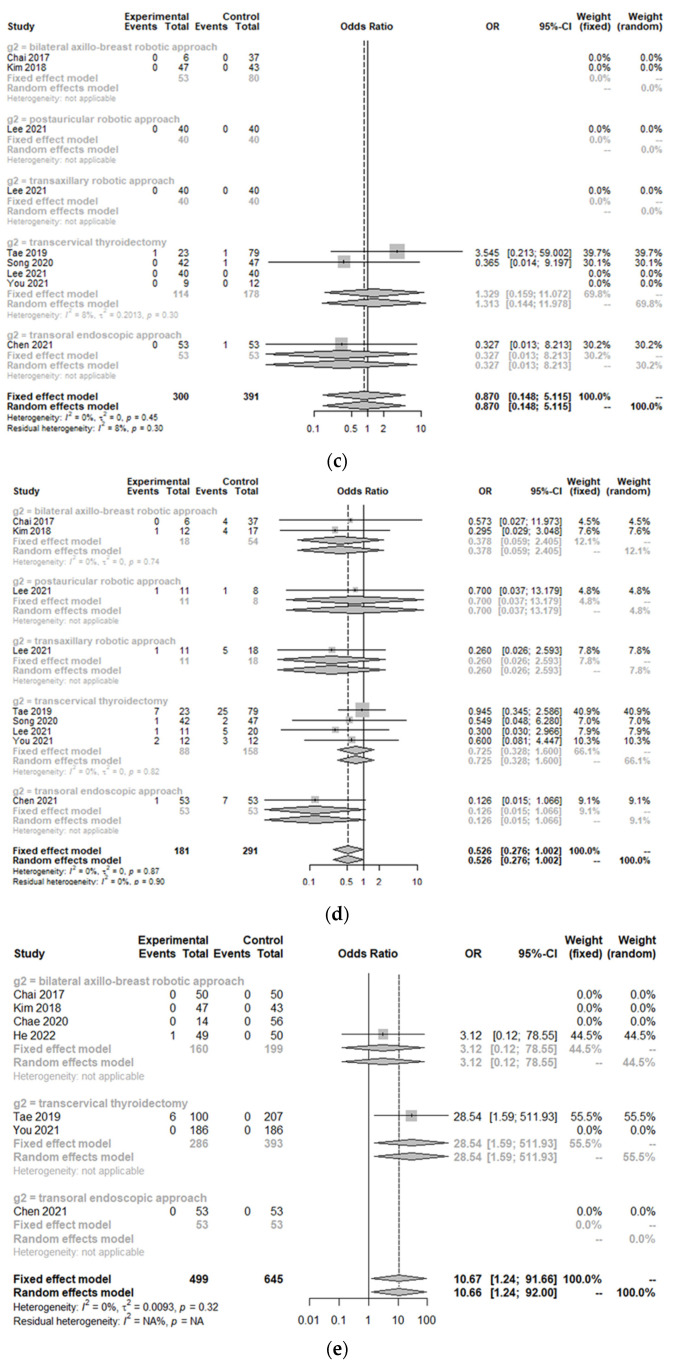

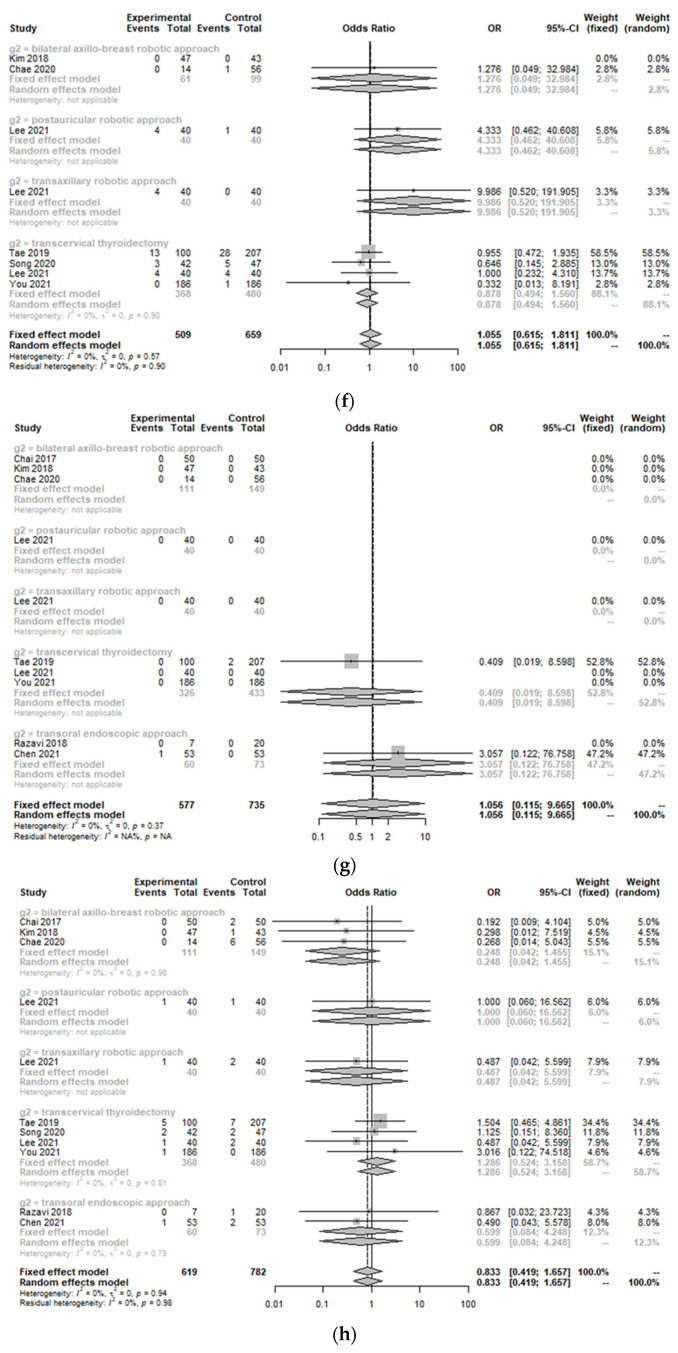
Forest plot for standard mean difference in complications (hematoma (**a**), flap perforation (**b**), permanent hypoparathyroidism (**c**), temporary hypoparathyroidism (**d**), postoperative infection (**e**), seroma (**f**), permanent vocal cord palsy (**g**), and temporary vocal cord palsy (**h**)) (total: number of participants per group).

Incidences of postoperative bleeding (hematoma) (OR 0.85, 95% CI [0.30; 2.45]; I^2^ = 0%), temporary and permanent hypoparathyroidism (OR 0.53, 95% CI [0.28; 1.00]; I^2^ = 0%/OR 0.87, 95% CI [0.15; 5.12]; I^2^ = 0%), temporary and permanent vocal cord palsy (OR 0.83, 95% CI [0.42; 1.66]; I^2^ = 0%/OR 1.06, 95% CI [0.12; 9.67]; I^2^ = 0%), and seroma (OR 1.06, 95% CI [0.62; 1.81]; I^2^ = 0%) were not significantly different between the TORT group and the control group (Figure 4). However, the postoperative infection rate (OR 10.67, 95% CI [1.24; 91.66]; I^2^ = 0.4%) was significantly higher in the TORT group than in the control group. There was no significant heterogeneity (I^2^ < 50%) in postoperative complications (Figure 4).

### 3.3. Postoperative Outcomes between the Treatment Group and Control Group

**Figure 5:**
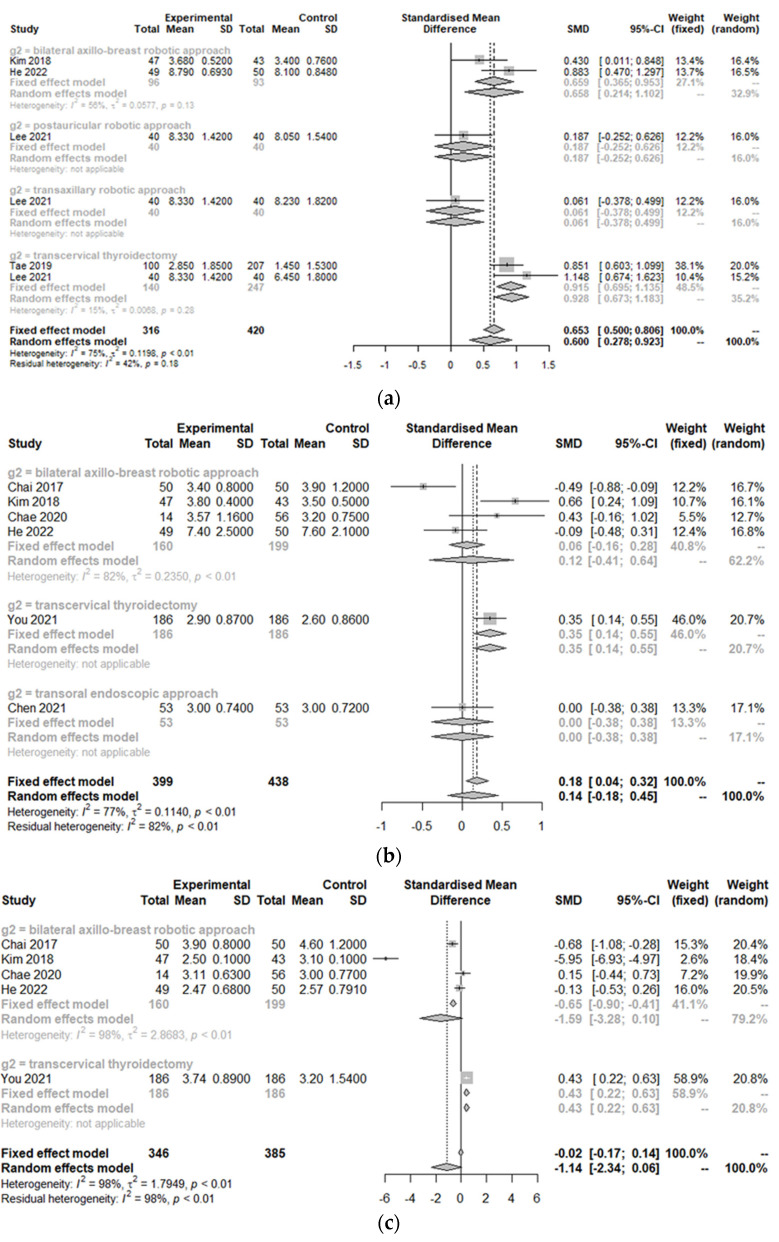

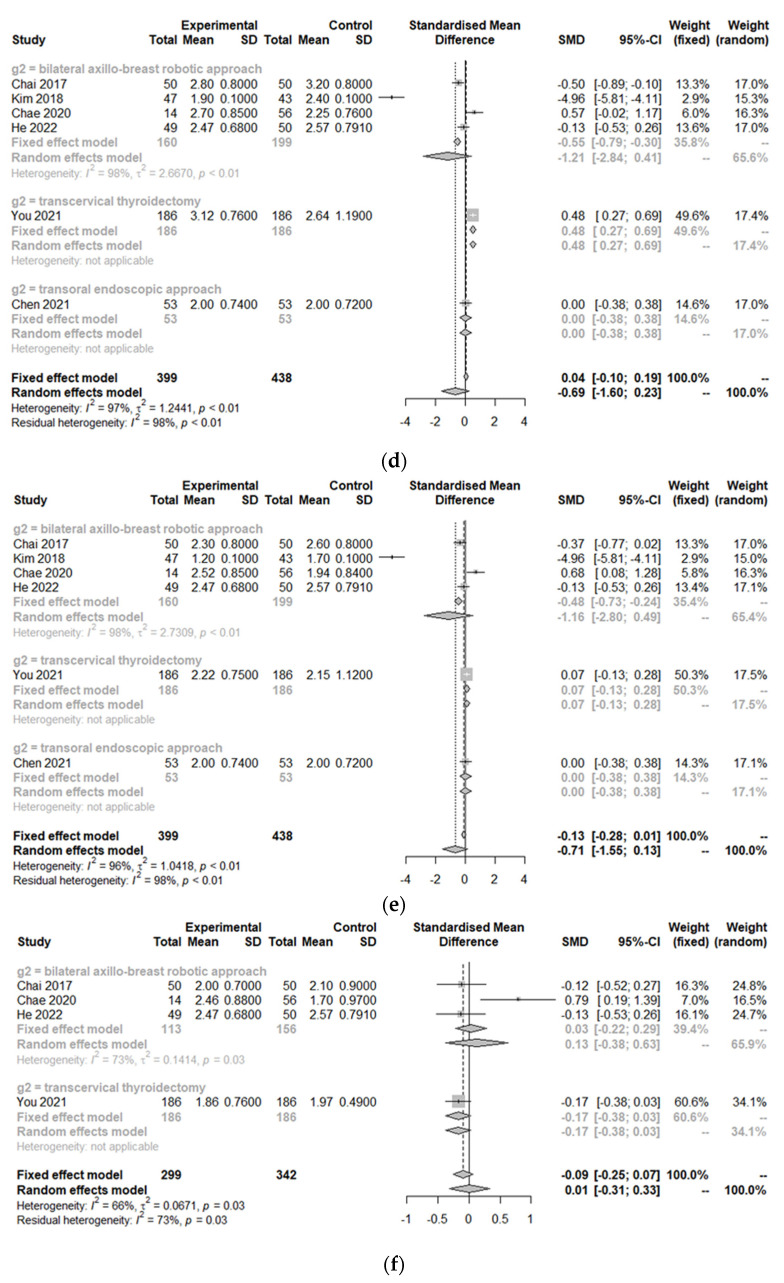
Forest plot for standard mean difference in postoperative outcomes (cosmetic satisfaction (**a**), hospitalization day (**b**), postoperative pain score on the operation day (**c**), postoperative day 1 (**d**), day 2 (**e**), day 3 (**f**)) (total: number of participants per group).

The postoperative cosmetic satisfaction score (SMD 0.60, 95% CI [0.28; 0.92]; I^2^ = 75%) was statistically higher in the TORT group than in the control group. However, there was no significant difference in postoperative pain score on op day (SMD −1.14, 95% CI [−2.34; 0.06]; I^2^ = 97.6%), at 1 day postoperation (SMD −0.68, 95% CI [−1.60; 0.23]; I^2^ = 96.9%), 2 days postoperation (SMD −0.71, 95% CI [−1.55; 0.14]; I^2^ = 96.4%), or 3 days postoperation (SMD 0.01, 95% CI [−0.31; 0.33]; I^2^ = 66.3%) or hospitalization days (SMD 0.14, 95% CI [−0.18; 0.45]; I^2^ = 76.8%) between the two groups (TORT and control) (Figure 5).

Because of significant heterogeneity (I^2^ > 50%) in postoperative outcomes, subgroup analysis was conducted according to the type of operation. There were no significant differences in postoperative outcomes (cosmetics, pain, or hospitalization) between robotic surgeries (TORT and robotic BABA). However, the TORT group had significantly higher patient satisfaction regarding cosmetic view (SMD 0.93, 95% CI [0.67; 1.18]). The TORT group also showed significantly increased hospitalization days (SMD 0.35, 95% CI [0.18; 0.51]) and postoperative pain on the operation day (SMD 0.43, 95% CI [0.22; 0.63]) and the first postoperative day (SMD 0.48, 95% CI [0.27; 0.69]) compared to the conventional OT group.

### 3.4. Sensitivity Analyses

Sensitivity analyses were performed to determine differences in integrated estimates in such a way that the meta-analysis was repeated, excluding one study each time. All results were consistent with the results described above.

## 4. Discussion

This meta-analysis compared operative outcomes and complications of TORT with other thyroid surgeries (robotic BABA, robotic PAA, conventional OT, and TOETVA). Many systematic reviews and meta-analyses have compared robot-assisted thyroid surgery, endoscopic thyroid surgery, conventional OT, and other approaches [2,33,34,35,36,37,38,39,40,41,42,43]. However, to the best of our knowledge, this meta-analysis is the first to directly compare TORT with other approaches for thyroid tumors.

In our study, the operation time and retrieved LN number were significantly different between the TORT and the control group. The TORT group also showed significantly longer operation times and fewer retrieved LNs than the other approaches in the subgroup analysis. In general, in other surgeries as well as thyroid surgery, the surgical time of a robotic surgery is longer than that of a conventional OT [44,45]. This is because a robotic-assisted surgery requires additional operation time to make flaps and dock the robotic arm and system to approach the thyroid tumor [29]. The fact that there were fewer retrieved LNs in the TORT group from our study might be because the clinician’s accumulated experiences for TORT and conventional OT differed during the same period, resulting in a selection bias [46]. Because TORT is more newly introduced than conventional OT, the overall proficiency of TORT might be lower than that of OT. Robotic lateral neck dissection by a gasless unilateral axillo-breast approach is possible. The retrieved LN number can be changed if the clinician has more experience with the robotic-assisted approach and becomes skilled [47]. In addition, the reason why TORT has fewer retrieved LNs than conventional OT is probably that the insertion site in the oral cavity is narrow, or the carbon dioxide gas expansion makes the workspace unstable. This working environment can limit the motion range of the robotic arm for LN dissection. The type of LN dissection can also affect the retrieved LN numbers. Kim et al. have reported that the unilateral or bilateral central neck dissection type can affect retrieved LN numbers in thyroid cancer [46]. The robot-assisted approach does not have an incision at the lower central part of the neck. Thus, the central neck dissection range and retrieved LN number might be lower than those of OT [46]. For example, in robotic BABA, the central LN may not be sufficiently exposed because it is blocked by the clavicle [48]. In TORT, central neck dissection exposure could vary depending on the inflexible robotic arm and the camera. However, even though the retrieved LN number has been reported as a good prognostic factor in gastric or colon cancer surgery [49,50], whether the retrieved LN number affects survival and recurrence in thyroid cancer surgery is currently unclear [46].

According to our meta-analysis, the TORT group had a significantly higher postoperative cosmetic satisfaction score than the control group. Another study has also confirmed that cosmetic satisfaction with the robot-assisted approach is significantly higher than with conventional OT [34]. In particular, transoral thyroidectomy is a natural orifice transluminal endoscopic surgery technique that leaves no scar on the neck or body. In addition, it is less invasive than other minimally invasive thyroid surgery [51]. Although many extra-cervical approaches to the thyroid have been described to avoid anterior neck scarring, such as trans-axillary, robotic face lift, and so on, these incisions are not truly scar-free. Therefore, TORT tends to be more cosmetically effective than robotic BABA, almost reaching statistical significance (SMD 0.66, 95% CI [0.21; 1.10], *p* = 0.13).

However, this approach also has its own demerits beside the cosmetic aspect. Because the craniocaudal view and the workspace between both mental nerves are narrow, flap elevation might be technically challenging [51]. This difficulty causes this approach to exclude patients with a history of neck surgery or over-prominent mandible [30]. Because of this limited view and approach, patients with a highly placed upper pole of the thyroid, prominent Zuckerkandl tubercle, advanced thyroid cancer with tracheal or esophageal invasion, and posterior extrathyroid extension should be avoided. Wound changes from the external body surface to the oral cavity can also lead to numbness or paresthesia of the lower lip and chin skin (due to mental nerve injury) and postoperative infection (due to oral flora) [30]. Based on these, the clinician needs to decide which approach is suitable for patients accordingly.

From our results, the TORT group had significantly longer hospital stays than the OT group. The pain was also significantly more severe on the day of surgery and the first postoperative day in the TORT group than in the OT group. Compared to OT, TORT has a wider flap elevation with a working space from the intraoral incision and unilateral axilla to thyroid tumor [5], resulting in increased pain and a longer hospital stay. In another meta-analysis, the anterior chest pain score of a robot-assisted approach in the first week after surgery was also significantly higher than that of OT [52].

This meta-analysis has some limitations. First, since not many studies have directly analyzed TORT and other approaches for thyroid tumor, only a few studies were included in this meta-analysis. Second, most of the included studies were retrospective in nature. There were no randomized controlled trials. Thus, there might be a selection bias or reporting bias. Third, the incidence of complications was low in most groups, making it difficult to identify differences between TORT and control groups. Fourth, there might be heterogeneity of postoperative outcomes because postoperative management, such as surgical site compression, postoperative diet, and analgesic for pain control, differed depending on the institute. Lastly, postoperative pain was not evaluated by subgroup analysis depending on the location, such as the neck or anterior chest.

## 5. Conclusions

This meta-analysis found that TORT improved patients’ cosmetic satisfaction but increased operative time, hospitalization days, and postoperative pain compared to conventional OT. However, since the number of studies included in this meta-analysis was small, conclusive confirmation of the clinical benefit of TORT needs to be studied further.

## Figures and Tables

**Table 1:** The characteristics of included studies.

Study	Design	Total Number of Patients (n)	Age of Patients with Robotic Approach (years, mean ± SD)	Sex (F/M)	Nation	Body Mass Index (kg/m^2^, mean ± SD)	Tumor Size (cm, mean ± SD)	Comparison	Control Group	Clinicopathology	Outcomes
Chai 2017	Retrospective review	100	41.2 ± 9.4	93/7	Korea	22.8 ± 62.6	1.1 ± 0.8	TORT	Robotic BABA	Benign, PTC	Operative time, postoperative pain (VAS), hospitalization day, number of retrieved LNs, hypoparathyroidism, VCP, infection
Kim 2018	Prospective comparative study	90	39.8 ± 10.7	83/7	Korea	NA	0.9 ± 0.5	TORT	Robotic BABA	Benign, PTC, follicular neoplasm	Operative time, cosmetic satisfaction, postoperative pain (VAS) hospitalization day, number of retrieved LNs, metastatic LN, incidental parathyroidectomy, VCP, seroma, infection
Razavi 2018	Retrospective review	27	41.3 ± 15.8	23/4	USA	28.3 ± 8.1	NA	TORT	Transoral endoscopic approach	TORT: Benign, NITNP, PTC Control: Benign, NITNP, PTC, Hürthle cell carcinoma	Operative time, VCP
Tae 2019	Retrospective review	307	45.5 ± 18.8	82/235	Korea	24.6 ± 3.7	NA	TORT	Conventional OT	TORT: Benign, PTC, follicular, medullary carcinoma Control: Benign, PTC, follicular carcinoma	Operative time, cosmetic satisfaction, number of retrieved LNs, metastatic LN, incidental parathyroidectomy, hypoparathyroidism, VCP, hematoma, seroma, infection, skin flap perforation
Chae 2020	Retrospective review	70	40.88 ± 9.80	9/61	Korea	23.60 ± 4.31	0.75 ± 0.35	TORT	Robotic BABA	PTC	Operative time, postoperative pain (VAS), hospitalization day, number of retrieved LNs, metastatic LN, incidental parathyroidectomy, VCP, seroma, infection
Song 2020	Prospective comparative study	89	44.0 ± 12.8	NA	Korea	24.1 ± 3.6	12.6 ± 12.1	TORT	Conventional OT	Benign, well differentiated carcinoma	Operative time, number of hypoparathyroidisms, VCP, seroma, hematoma
Chen 2021	Retrospective review	106	43.38 ± 12.84	17/89	Taiwan	23.6 ± 3.56	2.19 ± 1.734	TORT	Transoral endoscopic approach	Benign, indeterminate thyroid nodules, Graves’ disease, malignant or suspicious nodules	Operative time, postoperative pain (VAS), hospitalization day, number of retrieved LNs, metastatic LN, hypoparathyroidism, VCP, seroma, infection
Lee 2021	Retrospective review	160	46.48 ± 11.45	33/127	Korea	25.64 ± 3.79	1.02 ± 0.97	TORT	Robotic trans-axillary approach, robotic PAA	Follicular neoplasm, benign, differentiated thyroid carcinoma	Operative time, cosmetic satisfaction, postoperative pain (VAS), hospitalization day, number of retrieved LNs, hypoparathyroidism, VCP, seroma, hematoma
You 2021	Retrospective review	372	43.1 ± 10.74	68/304	Korea	23.5 ± 3.82	0.70 ± 0.50	TORT	Conventional OT	PTC	Operative time, postoperative pain (VAS), hospitalization day, number of retrieved LNs, metastatic LN, incidental parathyroidectomy, VCP, seroma, infection
He 2022	Prospective comparative study	99	44.6 ± 11.8	21/78	China	25.2 ± 14.2	3.50 ± 3.3	TORT	Robotic BABA	PTC	Operative time, cosmetic satisfaction, postoperative pain (VAS), hospitalization day, number of retrieved LNs, metastatic LN, infection

SD: standard deviation, NA: not available, TORT: transoral robotic thyroidectomy, BABA: bilateral axillo-breast approach, PTC: papillary thyroid carcinoma, NITNP: noninvasive thyroid neoplasm with papillary-like nuclear features, OT: open thyroidectomy, PAA: postauricular approach, VAS: visual analogue scales, VCP: vocal cord palsy, LN: lymph node.

**Table 2:** Subgroup analysis of operation-related measurements according to the comparison with other operative types.

Subgroups	Positive Lymph Node Number (SMD [95% CIs]; I^2^)	Incidence of Skin Flap Perforation (OR [95% CIs]; I^2^)	Incidental Parathyroidectomy (OR [95% CIs]; I^2^)
**Overall**	−0.0858 [−0.3441; 0.1725]; 66.7%	6.0165 [0.9318; 38.8464]; 0.0%	1.3392 [0.3981; 4.5049]; 0.0%
Robotic bilateral axillo-breast approach	**N = 2** 0.3221 [−0.6146; −0.0296]; 0.0%	**N = 2** 5.8977 [0.5962; 58.3455]; 0.0%	**N = 2** 1.3392 [0.3981; 4.5049]; 0.0%
Robotic postauricular approach			
Conventional open thyroidectomy	**N = 2** 0.0629 [−0.2508; 0.3766]; 75.0%	**N = 1** 6.2563 [0.2526; 154.9504]; NA	
Transoral endoscopic approach			
***p* value**	0.0785	0.9766	

SMD: Standardized mean difference, CI: Confidence interval, OR: Odds ratio, NA: Not available.
